# Characterization of Bruch's Membrane Formation in Human Fetal Retina and De Novo Membrane Synthesis by hPSC-Derived Retinal Pigment Epithelium

**DOI:** 10.1167/iovs.66.6.40

**Published:** 2025-06-11

**Authors:** Emily P. Lanning, Matthew J. Branch, Philippa Harding, Miriam Margari, Alexander J. Smith, Robin R. Ali, Rachael A. Pearson

**Affiliations:** 1 Ocular Cell and Gene Therapy Group, Centre for Gene Therapy and Regenerative Medicine, King's College London, Tower Wing, Guy's Hospital, London, United Kingdom

**Keywords:** retinal pigment epithelium (RPE), bruch's membrane (BrM), blood-retina barrier, human, retinal development, fetal, embryonic stem cells, differentiation, extracellular matrix (ECM)

## Abstract

**Purpose:**

Little is known about the development of Bruch's membrane (BrM), the structure separating and supporting the retina and choroid, nor whether differentiation of human pluripotent stem cell (hPSC)-derived retinal pigment epithelium (RPE) accurately replicates BrM. This has relevance for tissue engineering strategies, both in the development of accurate in vitro models, and effective RPE transplant strategies. Here, we investigated BrM-associated protein production in human fetal tissue and hPSC-derived RPE.

**Methods:**

The presence of laminin, elastin, fibronectin, and types I/III/IV collagen was examined in human fetal eyes at 6 to 21 post-conception weeks (PCWs) and hPSC-derived RPE cultures at 1 to 6 weeks in culture using immunohistochemistry/immunocytochemistry and quantitative PCR (qPCR).

**Results:**

In human fetal retina, laminin and fibronectin were present from 6 PCW, type IV collagen from 8 PCW, elastin from 12 PCW, type I collagen by 17 PCW, and type III collagen from 21 PCW. BrM layering was discernible from 12 PCW, becoming distinct by 17 PCW. In hPSC-derived RPE cultures, basement membranes containing laminin and fibronectin were present from week 1, type IV collagen from week 2, and type I collagen from week 4. Type III collagen was present at all timepoints, although not localized as a basement membrane. Elastin was absent at all timepoints.

**Conclusions:**

BrM-like membrane synthesis in hPSC-derived RPE largely recapitulates the temporal sequence seen in human development, excluding elastin. These support the utility of hPSC-derived RPE in in vitro systems to model RPE/retina interactions in health and disease, and inform cell therapy approaches, as de novo BrM-like membrane has the potential to support transplanted donor RPE.

Bruch's membrane (BrM) is an acellular layer produced by the retinal pigment epithelium (RPE) and the choroid, separating the retina and RPE from the choroid. It has many functions, primarily contributing toward retinal and choroidal health. One key role is to provide structural support for the RPE, a polarized monolayer of cells that, in turn, performs several essential tasks to maintain retinal health. Once fully developed, BrM is approximately 2 µm thick, and contains 5 layers: the basal lamina of the RPE, the inner collagenous layer (ICL), the elastic layer (EL), the outer collagenous layer (OCL), and the basal lamina of the choriocapillaris.^1^ These layers each have a distinct composition of several extracellular matrix (ECM) proteins, including laminins, collagens (types I, III, IV, V, VI, and VIII), fibronectin, fibrillin, and elastin^1^^–^^8^ (Fig. 1A). Although aging- and disease-related changes to BrM have been comprehensively studied,^6^^,^^9^^–^^15^ and BrM development has to a certain degree been examined in non-human mammals^16^^–^^19^ and chicks,^20^^,^^21^ very little is known about BrM development in humans.

**Figure 1.**
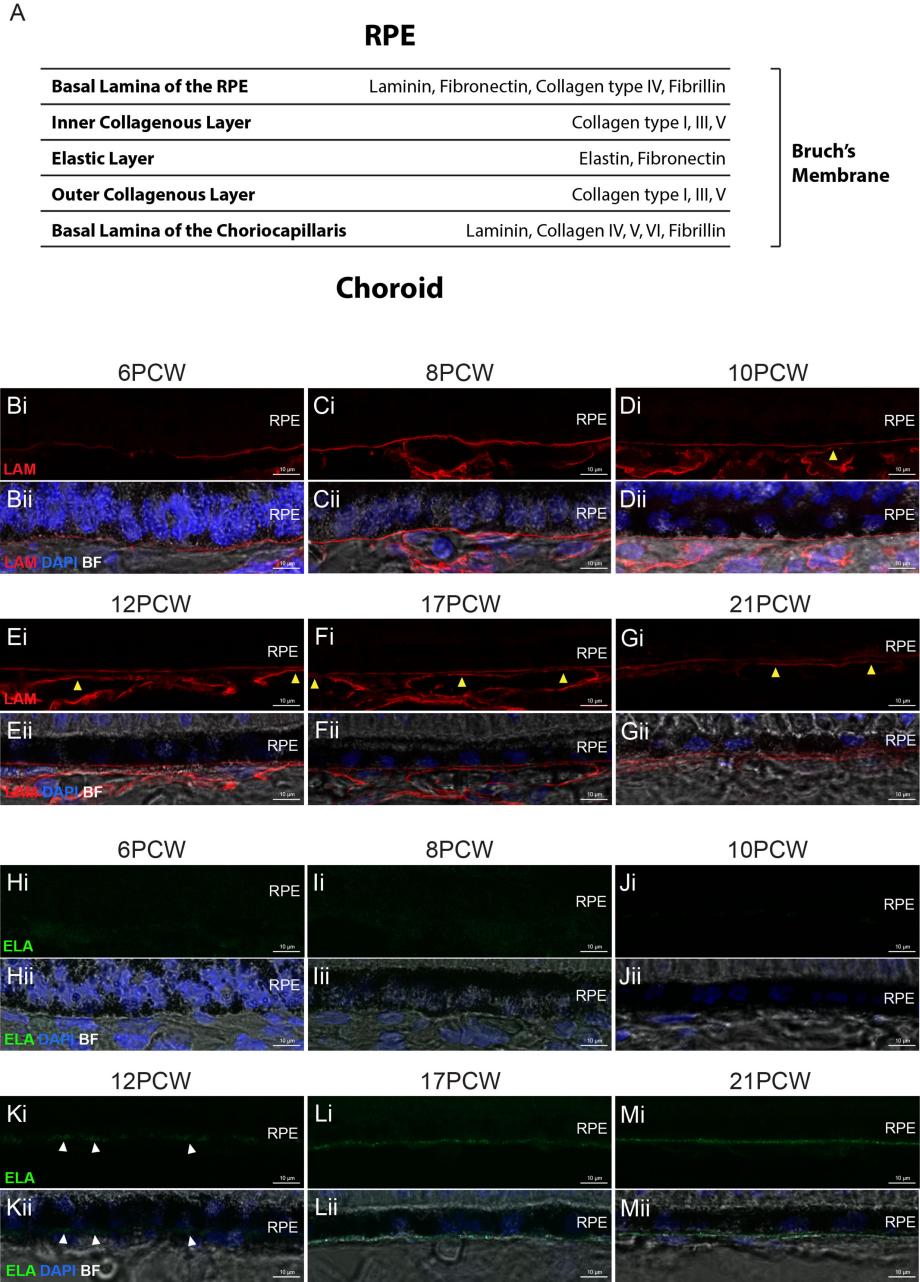
**Laminin and** **elastin protein deposition in PCW** **6–21 human fetal BrM.** (**A**) Simplified schematic of the layered structure of BrM, outlining some of the ECM proteins present in each layer.^1^^–^^8^ (**B**–**M**) Cryosections through human fetal retina immunolabeled with Laminin (LAM; *red*) and Elastin (ELA; *green*). (**B–G**) Immunostaining for laminin over time shows labeling as a defined line immediately basal to the RPE at all timepoints examined, in addition to a second layer detectable from 10 PCW (*yellow arrowheads*). (**H–M**) Immunostaining for elastin over time shows punctate signal immediately basal to the RPE from 12 PCW (*white arrowheads*) becoming more defined through development. Images show confocal maximum intensity projections (MIPs) of a z-stack approximately 1 µm thick, taken from the superior retina in the equatorial region. *Scale bars* = 10 µm. BF, Brightfield; BrM, Bruch’s membrane; PCW, post-conception weeks; RPE, retinal pigment epithelium. Nuclei were counter stained with DAPI (*blue*).

Many retinal diseases, most notably age-related macular degeneration (AMD), involve disruption of BrM and/or dysfunction and degeneration of the RPE, and a secondary loss of photoreceptor cells, leading to irreversible vision loss. Human pluripotent stem cells (hPSCs) have the potential to provide an unlimited source of healthy human RPE, which could be used for cell replacement therapy to treat diseases such as AMD. Moreover, with the use of induced pluripotential stem cells (iPSCs) from patients and gene editing techniques such as clustered regularly interspaced short palindromic repeats (CRISPR), hPSC-derived RPE offers the opportunity to model human diseases in vitro. Indeed, there is increasing interest in engineering more complex in vitro models of human ocular tissues to both probe disease mechanisms and develop therapeutic interventions.^22^

For RPE to avoid apoptosis and provide a barrier function and phagocytic capabilities, it must maintain adherence to a supporting substrate^23^ and remain as a polarized monolayer.^24^ Although there is evidence supporting the notion of BrM synthesis by hPSC-derived RPE in vitro, most published reports have examined one or two markers of BrM, and none have compared composition to human development. For example, hPSC-derived RPE cultured as monolayers in vitro has been reported to synthesize various ECM proteins, including laminin,^25^^–^^27^ type I collagen,^28^ type IV collagen,^25^^–^^27^^,^^29^^–^^32^ type VIII collagen,^30^ fibronectin,^27^^,^^31^^,^^32^ fibrillin,^31^ and elastin,^27^ and to form a de novo BrM-like basement membrane located basally to the RPE. Evidence suggests that this process may be accelerated when RPE is co-cultured with endothelial cells^27^^,^^28^^,^^31^^,^^32^ because specific angiocrine factors secreted by endothelial cells, including those of the choroid, are known to catalyze the crosslinking of collagens and elastins.^28^^,^^33^

Notably, replicating BrM formation by transplanted RPE has proven difficult to achieve through cell suspension transplants,^34^^–^^36^ prompting numerous attempts to transplant RPE as a pre-organized monolayer, with or without artificial support.^25^^,^^26^^,^^30^^,^^37^^–^^42^ The most comprehensively tested approach has been the transplantation of RPE grown on non-degradable scaffolds, which have progressed to clinical trials, but have been associated with adverse effects related to the need for prolonged immunosuppression.^37^ The presence of a permanent foreign object in the sub-retinal space may also increase the risk of retinal detachment and/or scarring, raising interest in degradable scaffolds that could be replaced by de novo ECM upon degradation of the artificial scaffold.

To develop better in vitro RPE models of health and disease and to assist with designing optimal RPE transplantation strategies, it is therefore important to understand the in vivo development of BrM and how it compares to synthesis by hPSC-derived RPE in vitro. This will help inform if, and what, additional support may be required to accompany RPE cells in both disease modeling and transplantation scenarios. Here, we investigated the synchronicity of BrM-associated ECM protein synthesis in human fetal tissue and hPSC-derived RPE.

## Methods

### Human Tissue Preparation

Human fetal eyes were obtained from the Human Developmental Biology Resource (HDBR) tissue bank (http://www.hdbr.org/) with ethical approval and informed consent (project number: 200599). This study adhered to the tenets of the Declaration of Helsinki. All samples were the result of elective termination of pregnancies. Karyotyping revealed that no tissue included in this study had any chromosomal abnormalities and visual checks were performed by trained HDBR staff for normal development. Fetal eyes (*n* = 1 per timepoint) from 6, 8, 10, 12, 17, and 21 post conception weeks (PCWs) were included in this study. Fetal eyes/heads were dissected and fixed in 10% formalin for 24 hours before storage in PBS for cold transportation. Upon arrival, samples were stored in PBS at 4°C until ready for use. If necessary, samples were further dissected to remove excess tissue, then cryopreserved in 20% sucrose overnight at 4°C. Whole eyes were embedded in O.C.T. (TissueTek) and 12 µm sections were cut onto Superfrost slides using a cryostat. Slides were stored at −80°C.

### Immunohistochemistry

Fetal tissue slides were removed from −80°C storage and left to thaw for 30 minutes at room temperature (RT). Tissue was permeabilized and blocked using PBS containing 0.1% Triton, 2% BSA, and 5% donkey serum for 1 hour at RT. Slides were incubated with primary antibodies overnight at 4°C. Primary antibodies were diluted in PBS containing 0.1% Triton and 2% BSA, and the specific antibodies and dilutions used are detailed in Supplementary Table S1. Slides were washed with PBS and incubated in secondary antibodies (Alexa Fluor, 1:200, ThermoFisher, UK) for 1 hour at RT. Slides were washed and nuclei were stained with 4′,6-Diamidino-2-Phenylindole (DAPI; 300 nM) for 10 minutes at RT. Slides were mounted with coverslips using fluorescence mounting medium (Dako, Denmark), wrapped to protect them from light, and stored at 4°C. Negative controls were prepared as above but with the absence of a primary antibody. For comparison between samples, all images were taken at the equatorial point, superior of the optic nerve, near the point of insertion of the superior rectus muscle, unless otherwise stated. Fluorescent and brightfield images were acquired using a ZEISS LSM 900 confocal microscope. Images were processed using the image processing software, ImageJ (Fiji). Brightness and contrast were adjusted equally across all timepoints for each individual marker to allow for comparison and the visualization of localization of proteins.

### hPSC-Derived RPE Culture

The human embryonic stem cell (hESC) line H9 (WiCell, Madison, WI, USA) was maintained on vitronectin (ThermoFisher, UK) coated tissue culture plates in mTeSR Plus medium (STEMCELL Technologies, Canada). Cells were passaged every 3 to 4 days and media was changed daily. Cells were allowed to grow to approximately 95% confluency before differentiation was initiated following our previously reported protocol^43^ with some minor modifications. Patches of pigmented RPE appeared in the cultures from 4 weeks of differentiation and were manually dissected from the cultures between 4 and 8 weeks of differentiation and enzymatically dissociated. Cells were resuspended in media containing DMEM/F12, 2% B27 and antibiotic-antimycotic (all ThermoFisher, UK), supplemented with 10% human platelet lysate (hPL; Sexton Biotechnologies, USA) and plated on vitronectin coated tissue culture plates. Media was changed every 2 to 3 days and hPL was gradually reduced over 2 weeks. RPE cells proliferated until confluent and were subsequently passaged and their numbers expanded. After the RPE was passaged for a second time, it was seeded onto vitronectin-coated glass coverslips for immunohistochemistry/quantitative PCR (qPCR) or ThinCerts (Greiner Bio-One, UK) for analyzing growth factor secretion. The time elapsed since seeding after the second passage is denoted as weeks of differentiation. For each passage, the day of passage and cell seeding is considered day 0.

### Quantitative Reverse Transcription Polymerase Chain Reaction 

The hPSC-derived RPE cells were lysed at 2-, 4-, and 6-week timepoints post-seeding, and RNA extracted using RNeasy Micro Kit (QIAGEN, UK), as per the manufacturer's instructions. Samples were eluted in 14 µl and concentration and RNA quality analyzed using a BioDrop spectrophotometer. The cDNA synthesis was conducted using QuantiTect Reverse Transcription Kit, according to the manufacturer's instructions. Quantitative RT-qPCR was conducted using Perfecta LowROX Mastermix with custom primers listed in Supplementary Table S2 from Integrated DNA Technologies. All samples were run in triplicate alongside two endogenous reference genes (*GAPDH* and *ACTB*). Water and no-RT negative controls were run alongside samples. Comparative Ct was calculated to determine relative gene expression as fold change from undifferentiated PSCs using the mean of the two reference genes.

### Immunocytochemistry

The hESC-derived RPE grown on coverslips was fixed using 4% PFA, before washing with PBS. Cells were permeabilized and blocked (0.1% Triton, 1% BSA, and 5% donkey serum) for 1 hour at RT. Coverslips were incubated with primary antibodies overnight at 4°C. Primary antibodies were diluted in PBS (0.1% Triton and 1% BSA) and the specific antibodies and dilutions used are detailed in Supplementary Table S1. Cells were washed with PBS and incubated in secondary antibodies (Alexa Fluor, 1:200, ThermoFisher, UK) for 1 hour at RT. Cells were washed and nuclei were stained with DAPI (1:1000) for 10 minutes at RT. Coverslips were mounted on slides using fluorescence mounting medium (Dako, Denmark), and stored at 4°C and protected from light. Negative controls were prepared as above, but with the absence of a primary antibody. Fluorescent and brightfield images were acquired using a ZEISS LSM 900 confocal microscope. Images were processed using the image processing software, ImageJ (Fiji).

### Statistics and N Numbers

Relative mRNA expression of the target gene was calculated using the comparative Ct (2^ΔΔCt^) method,^44^ normalized to *GAPDH/ACTB* and relative to the levels of undifferentiated hPSCs for each target gene. This was chosen as one representing pluripotent cells prior to the initiation of differentiation into RPE. Mean, standard deviation (SD) and statistics were calculated from the ΔCt. All means are stated ±SD.

Statistical tests were performed using Graph Pad Prism version 10 software. The qPCR data were analyzed based on three samples from three independent differentiation batches per timepoint. One-way ANOVA with Dunnett's multiple-comparison test was used to assess the differences in gene expression across time relative to hPSCs. Statistical significance is presented in the figures as follows: *P < 0.05, **P < 0.01, ***P < 0.001, and ****P < 0.0001, ns = not significant.

Immunohistochemical expression analysis is based on representative findings and no quantitative assessments were performed. Human fetal samples comprised one per time point (additional samples were not possible given the scarcity of tissue) and conclusions based on immunohistochemical labeling were from at least three sections/sample. For hPSC-derived RPE immunocytochemistry, conclusions were drawn based on three independent differentiation batches, encompassing three coverslips per time point.

## Results

### Localization of BrM-Associated Proteins in Human Fetal Retina

Human fetal sections spanning 6 timepoints from 6 to 21 PCWs were assessed for the presence of 6 known BrM markers present in the 5 layers of the adult BrM: laminin, elastin, fibronectin, and types I, III, and IV collagens (see Fig. 1A). This period of development encompasses the majority of retinal neurogenesis.^45^^,^^46^ RPE cells are born around 5 PCW^47^ and are just beginning to pigment at 6 PCW (Fig. 1B). A standardized location in the superior retina, near the equator of the eye, was used unless otherwise stated, to account for the center-to-periphery gradient of retinal maturation.

**Figure 2.**
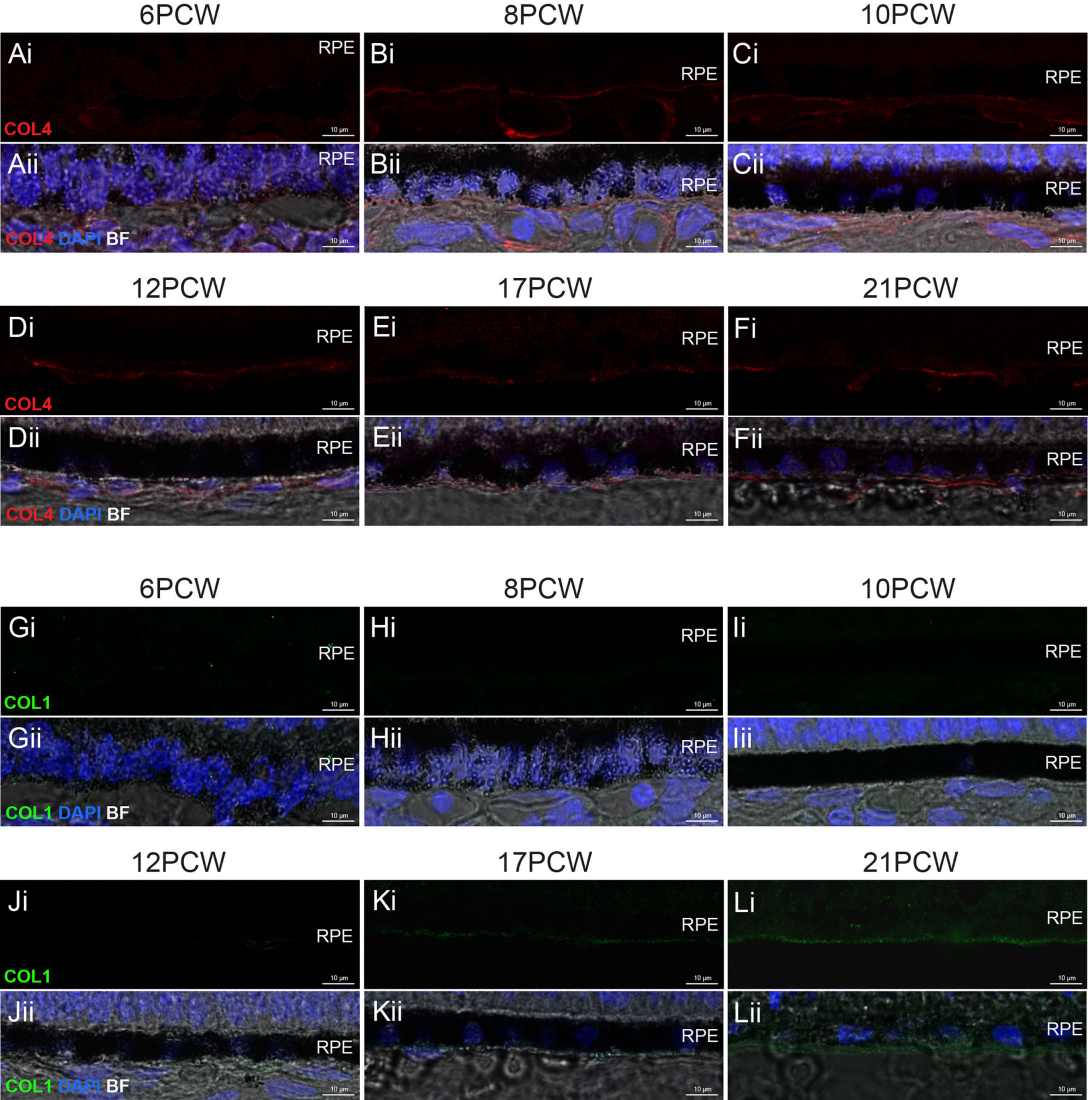
**Types IV and I** **collagen expression in PCW** **6–21 human fetal BrM.** Cryosections through human fetal retina and immunolabeled for type IV and I collagens. (**A–F**) Immunostaining for type IV collagen (COL4; *red*) over time shows labeling as a defined line immediately basal to the RPE from 8 PCW and all subsequent timepoints. (**G–L**) Immunostaining for type I collagen (COL1; *green*) over time reveals labeling basal to the RPE from 17 PCW. Images show confocal MIPs of a z-stack approximately 1 µm thick, taken from the superior retina in the equatorial region. *Scale bar* = 10 µm. BF, Brightfield; BrM, Bruch’s membrane; PCW, post-conception weeks; RPE, retinal pigment epithelium. Nuclei were counter stained with DAPI (*blue*).

**Figure 3.**
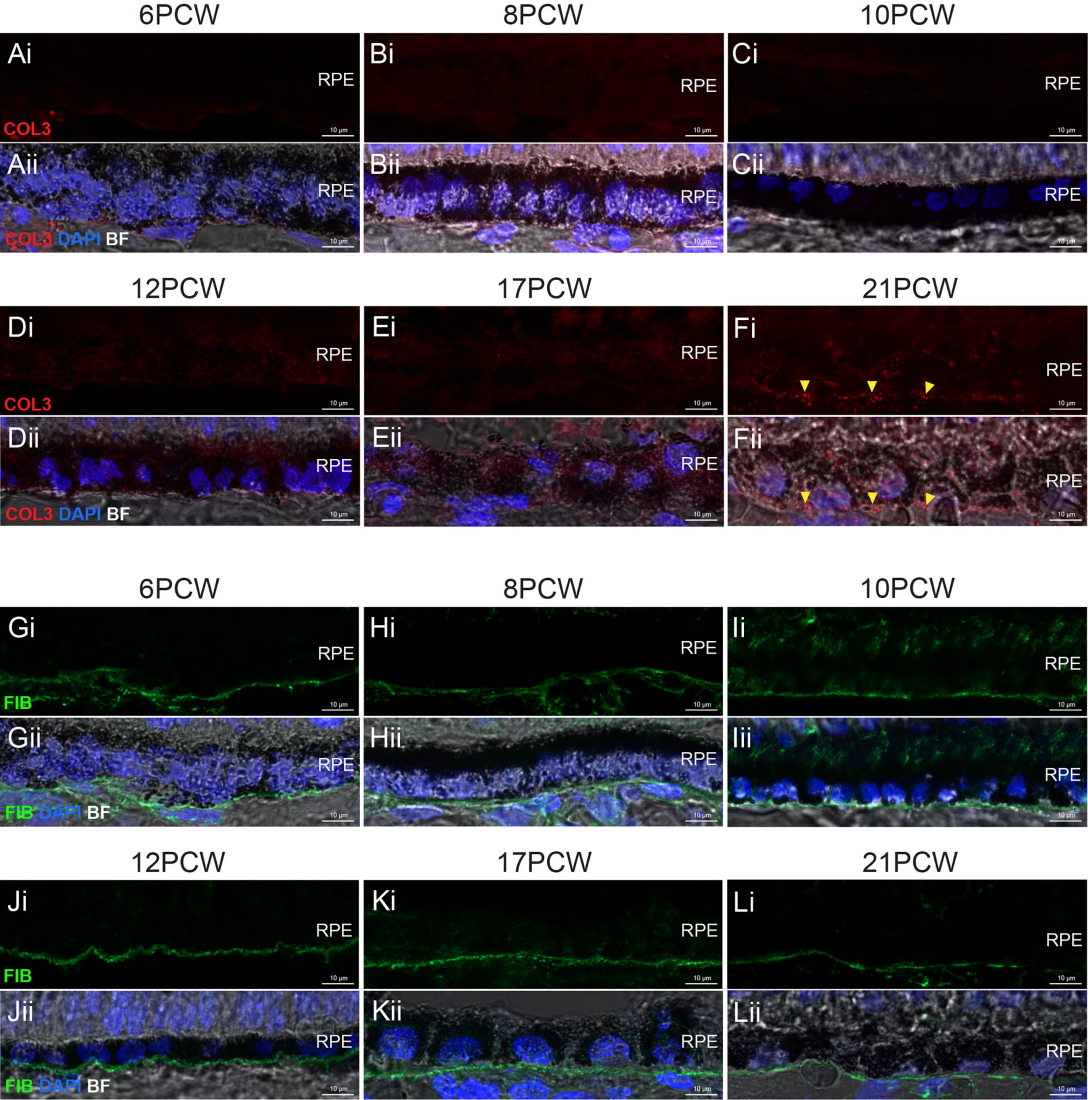
**Type III** **collagen and** **fibronectin expression in PCW** **6–21 human fetal BrM.** Cryosections through human fetal retina and immunolabeled for type III collagen and fibronectin. (**A–F**) Immunostaining for type III collagen (COL3; *red*) over time shows punctate labeling immediately basal to the RPE at 21 PCW only. (**G–L**) Immunostaining for fibronectin (FIB; *green*) over time shows labeling as a defined line immediately basal to the RPE at all timepoints examined. Images show confocal MIPs of a z-stack approximately 1 µm thick, taken from the superior retina in the equatorial region. *Scale bar* = 10 µm. BF, Brightfield; BrM, Bruch’s membrane; PCW, post-conception weeks. RPE, retinal pigment epithelium. Nuclei were counter stained with DAPI (*blue*).

A single, uninterrupted laminin^+ve^ layer localized to the nascent BrM was already present at PCW 6 and observed at all timepoints assessed (Figs. 1B–G). A second interrupted laminin^+ve^ layer, located approximately 1 to 2 µm below the first, was first discernible from 10 PCW and labeling of this layer became increasingly clear in later timepoints (see Figs. 1D–G, yellow arrowheads). Elastin was absent during the first 2 months of development, but a weak, punctate signal, located basal to the RPE, was first detected from 12 PCW (Figs. 1H–K, white arrowheads). Immunolabeling for elastin was stronger and more consistent by 17 PCW and presented as a clear, uninterrupted line at 21 PCW (Figs. 1L, 1M). No type IV collagen signal was detected at 6 PCW but was evident from 8 PCW onward (Figs. 2A–F). Immunolabeling for type I collagen staining was not visible before 12 PCW but was evident as a distinct layer by 17 PCW (Figs. 2G–L). Type III collagen was absent throughout early development, but a punctate signal was detected in the presumptive BrM only at 21 PCW (Figs. 3A–F, yellow arrowheads). Like laminin, fibronectin was seen at all timepoints assessed and was located basal to the RPE (Figs. 3G–L). It should be noted that laminin, elastin, types I, III, and IV collagens, and fibronectin are also found in the choroid,^48^ and were detected here, in the forming choroid. No fluorescent signal was detected in negative controls (Supplementary Fig. S1). Together, these results show that the temporal order in which these six BrM-associated proteins are expressed in human fetal BrM is fibronectin/laminin, type IV collagen, elastin, type I collagen, and, last, type III collagen.

As noted above, retinal maturation exhibits a center-to-periphery gradient. We therefore looked to see if this was evident in the expression of BrM proteins. Fibronectin and laminin are already robustly expressed at the earliest timepoint examined (PCW 6). However, type IV collagen is absent in the presumptive BrM at PCW 6, and present in PCW 8, in the equatorial region (see Figs. 2A, 2B) and we looked to see if a developmental gradient of expression could be detected. At 6 PCW, type IV collagen was present in neither the developmentally more mature center nor the periphery, whereas at 8 PCW, type IV collagen was already visible in both locations with no notable differences in expression in the two locations (Supplementary Fig. S2); this suggests that the onset of type IV collagen expression occurs quite rapidly between these two timepoints across the retina and interim timepoints would be required to discern any center-periphery gradients. Other markers were not examined due to larger time intervals between samples when they are expressed.

### Identification of Distinct BrM Layers in Human Fetal Retina

**Figure 4.**
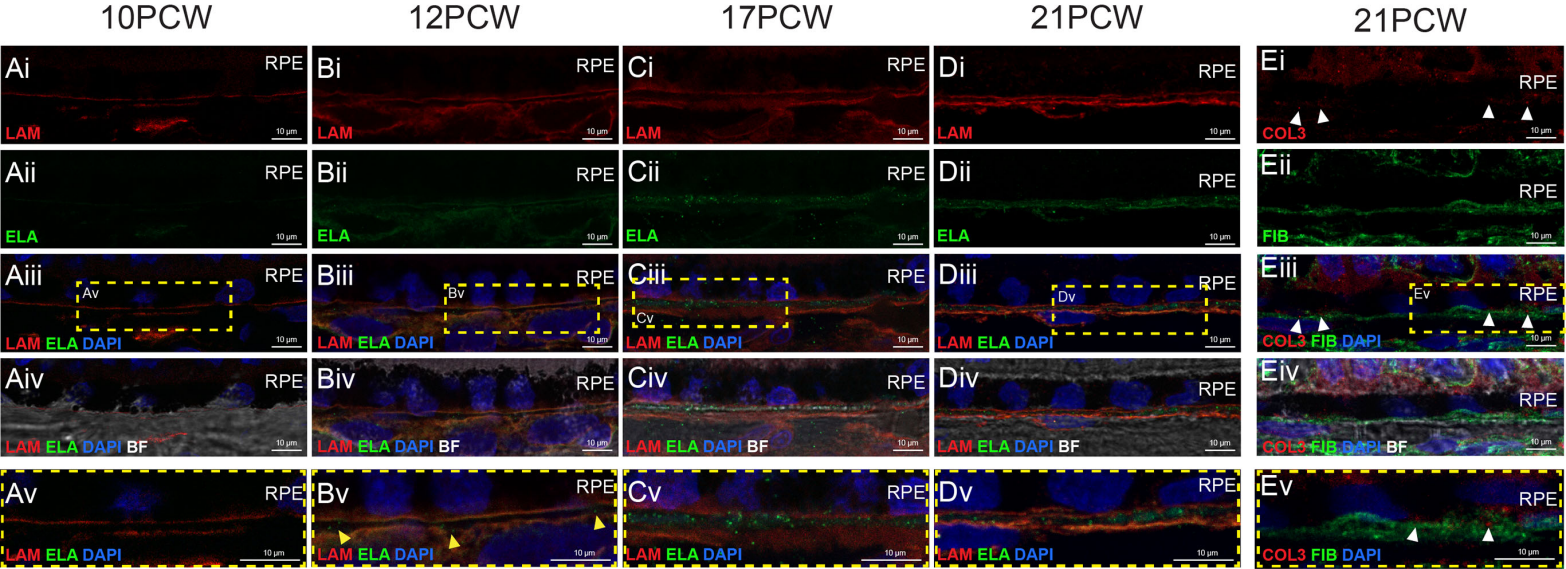
**Formation of the internal layers of BrM in human fetal retinal development.** Cryosections through human fetal retina and immunolabeled for (**A–D**) laminin (LAM; *red*) and elastin (ELA; *green*) and (**E**) type III collagen (COL3; *red*) and fibronectin (FIB; *green*) in (**A**) 10 PCW, (**B**) 12 PCW, (**C**) 17 PCW, and (**D,** **E**) 21 PCW eyes. (**Av–Ev**) Shows digital zoom of the ROI highlighted in (iii), *yellow box*. Images show confocal MIPs of a z-stack approximately 1 µm thick, taken from the superior retina in the equatorial region. *Scale bar* = 10 µm. Note: Despite careful control of pinhole size and sequential laser scanning, we observed some bleed-through from the very strong laminin signal (red channel) visible in the green channel. BF, Brightfield; BrM, Bruch’s membrane; PCW, post-conception weeks; RPE, retinal pigment epithelium. Nuclei were counter stained with DAPI (*blue*).

A key characteristic of mature BrM is the presence of five separate layers, with laminin present only in the outermost layers of BrM and elastin present only in the central layer (see Fig. 1A).^2^^,^^4^ Co-staining of these two markers showed two separate laminin^+ve^ layers (spaced approximately 1–2 µm apart) present from 10 PCW; however, no elastin was observed between these layers (Fig. 4A), which is in line with the observed appearance of elastin at 12 PCW (see Figs. 1H–K). Similar observations were made at 12 PCW, with the addition of some punctate elastin^+ve^ staining visible between the laminin^+ve^ layers (Fig. 4B, yellow arrowheads). By 17 PCW and 21 PCW, a robust elastin^+ve^ signal was consistently detected between the two lLaminin^+ve^ layers (Figs. 4C, 4D).

Similarly, in adult BrM, fibronectin is found in the elastic layer, in addition to the outermost layers. The elastic layer splits the two collagenous layers, whereas type III collagen is localized. As described above, type III collagen is not seen until PCW 21. We therefore co-stained PCW 21 sections for fibronectin and type III collagen to examine the establishment of these inner layers (Fig. 4E). Fibronectin was present as a continuous layer, and, in some regions, potentially as two layers. However, labeling for type III collagen deposition at this timepoint is still quite punctate, indicating that the collagenous layers are likely not yet fully developed, and it therefore is not possible to discern separate collagenous and elastic layers at this timepoint using these markers.

### Characterization of hPSC-Derived RPE

We next assessed BrM deposition by hPSC-derived RPE. To ensure hPSCs correctly differentiate into RPE in our hands, cultures were characterized prior to assessing ECM protein synthesis. The hPSC-derived RPE was grown on vitronectin-coated glass coverslips and assessed weekly using brightfield microscopy; this revealed morphological features typical of native RPE, including increasing pigmentation and “cobblestone” morphology from 3 weeks post-seeding (Supplementary Figs. S3A–F). Immunostaining of hPSC-derived RPE after 6 weeks revealed the presence of adherens junctions between cells (ZO-1), apical microvilli (Ezrin), and other mature RPE markers, including bestrophin1 and RPE65 (Supplementary Fig. S3G–K). RPE genes *BEST1*, *MITF*, and *RPE65* were upregulated in hPSC-derived RPE at 2, 4, and 6 weeks in culture, relative to undifferentiated hPSCs (Supplementary Figs. S3L–N). Basolateral secretion of VEGF was significantly higher than apical VEGF secretion (Supplementary Fig. S3O), and ezrin staining was localized to the apical surface of the cells (Supplementary Fig. S3K), indicating that the cells had developed into a polarized RPE monolayer, similar to native RPE.^49^

### Relative Expression of ECM Genes in hPSC-Derived RPE In Vitro

**Figure 5.**
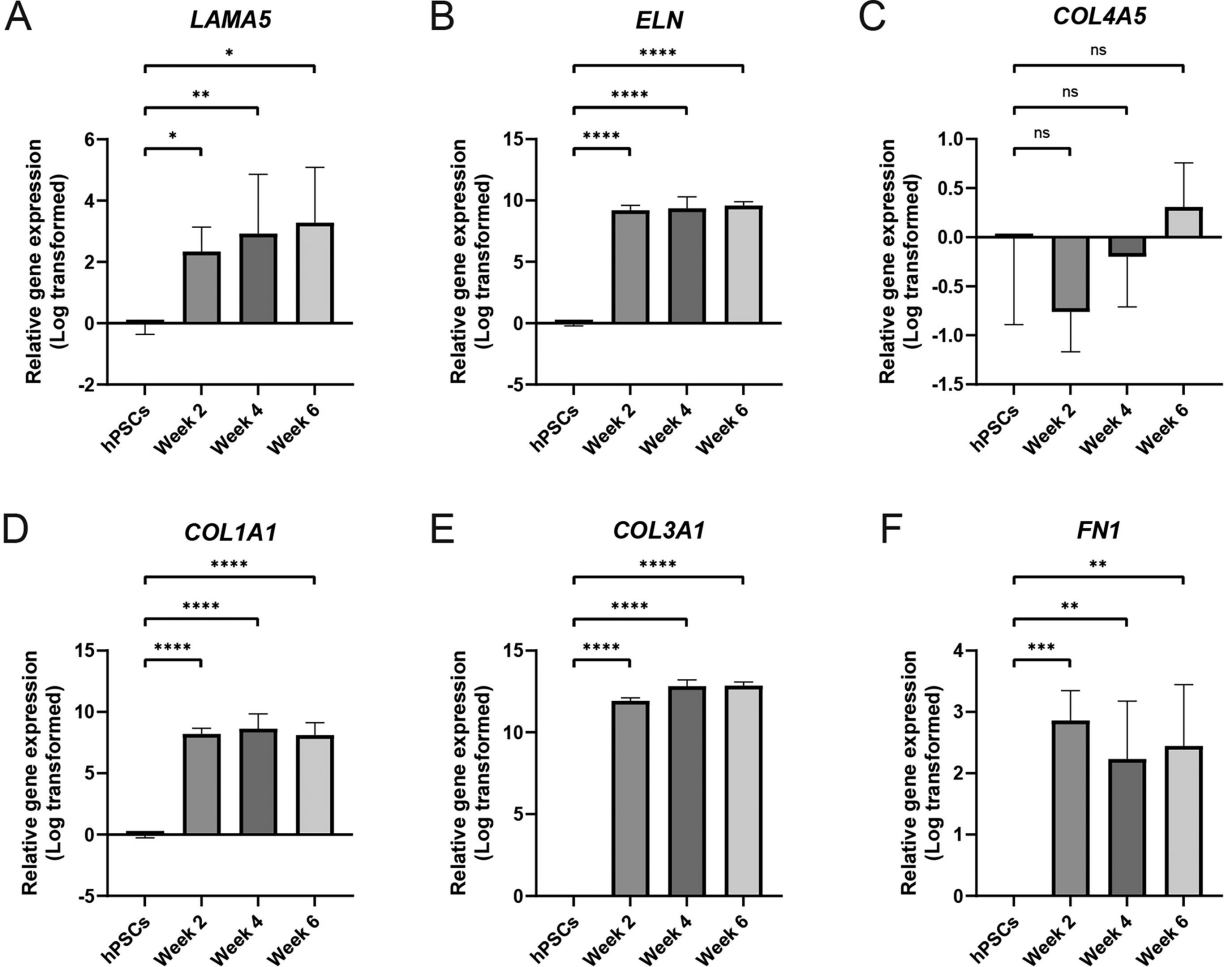
**ECM genes are upregulated in hPSC-RPE relative to undifferentiated hPSCs.** (**A–F**) Relative gene expression analysis of *LAMA5*, *ELN*, *COL4A5*, *COL1A1*, *COL3A1*, and *FN1* in hPSC-RPE shows upregulation at weeks 2, 4 and 6, relative to undifferentiated hPSCs in *LAMA5*, *ELN*, *COL1A1*, *COL3A1*, and *FN1* but no significant difference in *COL4A5* expression, although Ct values indicate expression at all timepoints including undifferentiated cells (see Supplementary Fig. S3). One-way ANOVA with Dunnett's multiple-comparison test, *P < 0.05, **P < 0.01, ***P < 0.001, ****P < 0.0001.

Next, we assessed the relative gene expression of BrM-associated *LAMA5*, *ELN*, *COL1A1*, *COL3A1*, *COL4A5*, and FN1 in hPSC-derived RPE at 2-, 4-, and 6-weeks post-seeding. There was a significant upregulation in *COL1A1*, *COL3A1*, *ELN*, *FN1*, and *LAMA5* genes at all timepoints assessed, relative to undifferentiated hPSCs (Figs. 5A–F, Supplementary Fig. S4). Comparative Ct analysis showed no significant difference in *COL4A5* expression between undifferentiated hPSCs and hPSC-derived RPE (see Fig. 5C). However, undifferentiated hPSCs are known to express *COL4A5*,^50^ and cycle threshold values of between 28 and 30 indicated that *COL4A5* was expressed in all samples (see Supplementary Fig. S4C).

### Synthesis of BrM-Associated Proteins by hPSC-Derived RPE In Vitro

**Figure 6.**
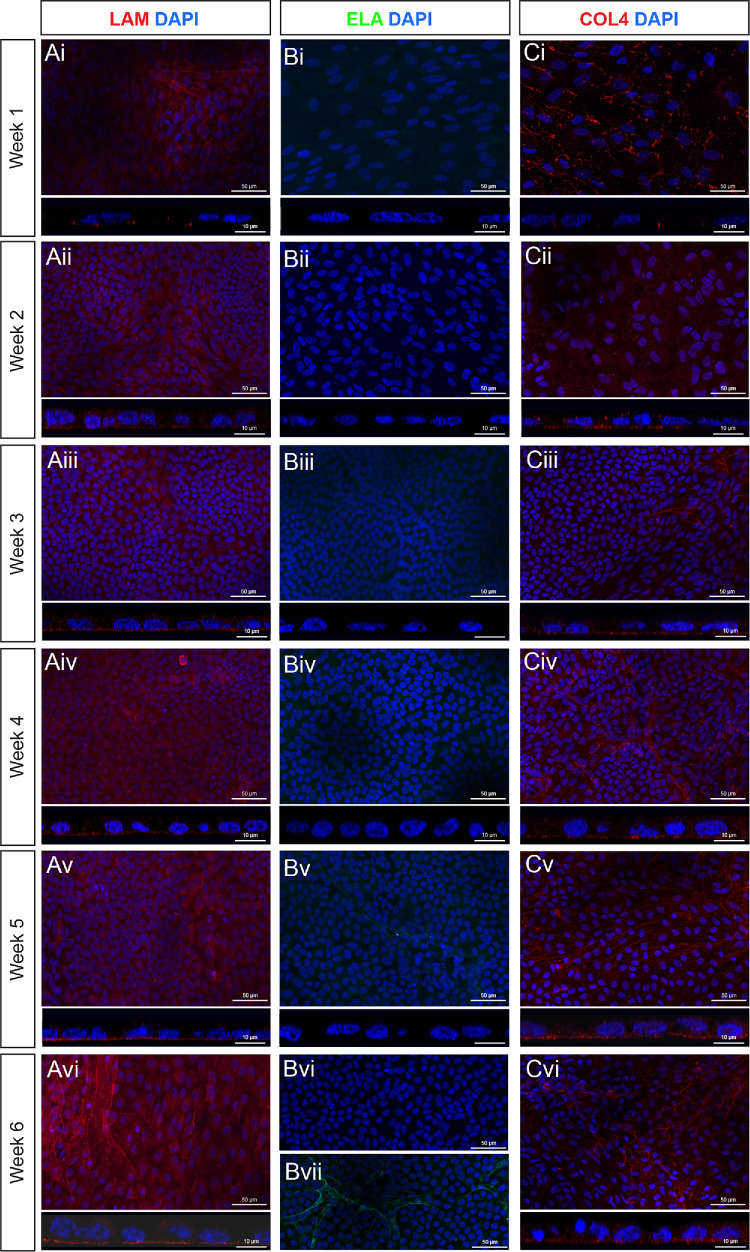
**ECM proteins** **laminin and** **type IV** **collagen are present from 1 week of hPSC-derived RPE differentiation,** **whereas elastin protein is not consistently** **synthesized** **de novo.** (**A–C**) Immunostaining for (**A**) laminin (LAM; *red*), (**B**) elastin (*ELA*; *green*), and (**C**) type IV collagen (COL4, *red*) over a 6-week culture period. Each panel shows representative xy confocal MIPs of a z-stack approximately 15 µm thick (*above*), alongside simulated cross-sections using xz orthogonal projections (*below*). Images show laminin labeling present from week 1 onward, and as a defined basement membrane located basally to the monolayer. Elastin labeling was absent in all representative images across all timepoints assessed, except for (**Bvii**) a single occurrence of elastin labeling was detected at the week 6 timepoint. Scattered type IV collagen labeling was present from week 1, becoming a defined basement membrane located basally to the monolayer from week 2 onward. Nuclei are counter stained with DAPI (*blue*).

**Figure 7.**
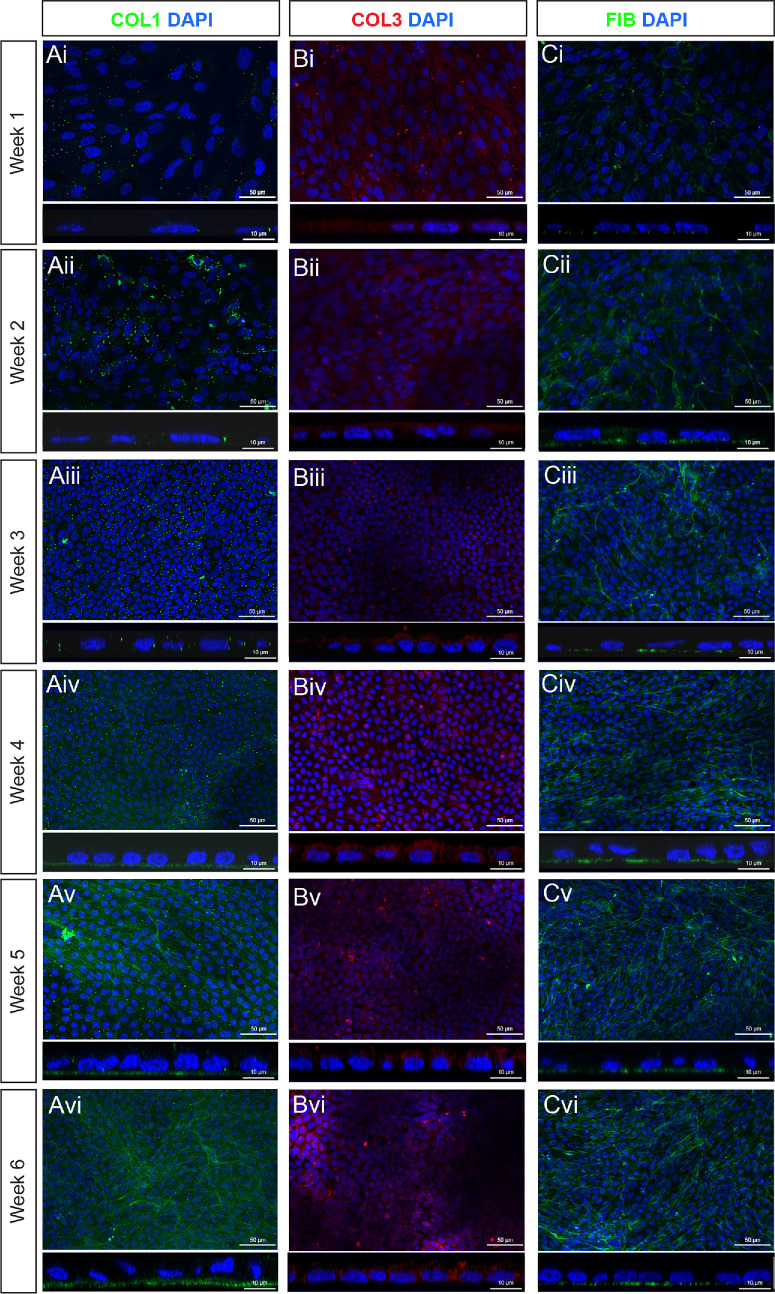
**ECM proteins** **type I** **collagen and** **fibronectin are present in the form of a basement membrane from 1 week of hPSC-derived RPE differentiation,** **whereas type III** **collagen protein is not deposited basally.** (**A–C**) Immunostaining for (**A**) type I collagen (COL1; *green*), (**B**) type III collagen (COL3; *red*), and (**C**) fibronectin (FIB, *green*) over a 6-week culture period. Each panel shows representative xy confocal MIPs of a z-stack approximately 15 µm thick (*above*), alongside simulated cross-sections using xz orthogonal projections (*below*). Images show type I collagen labeling present from week 1 as punctate deposits, becoming a defined basement membrane located basally to the monolayer from week 4 onward. Type III collagen signal was present in all timepoints throughout the full depth of the monolayer rather than as a basement membrane. Fibronectin labeling was present from week 1 onward, and as a defined basement membrane located basally to the monolayer. Nuclei are counter stained with DAPI (*blue*).

**Figure 8.**
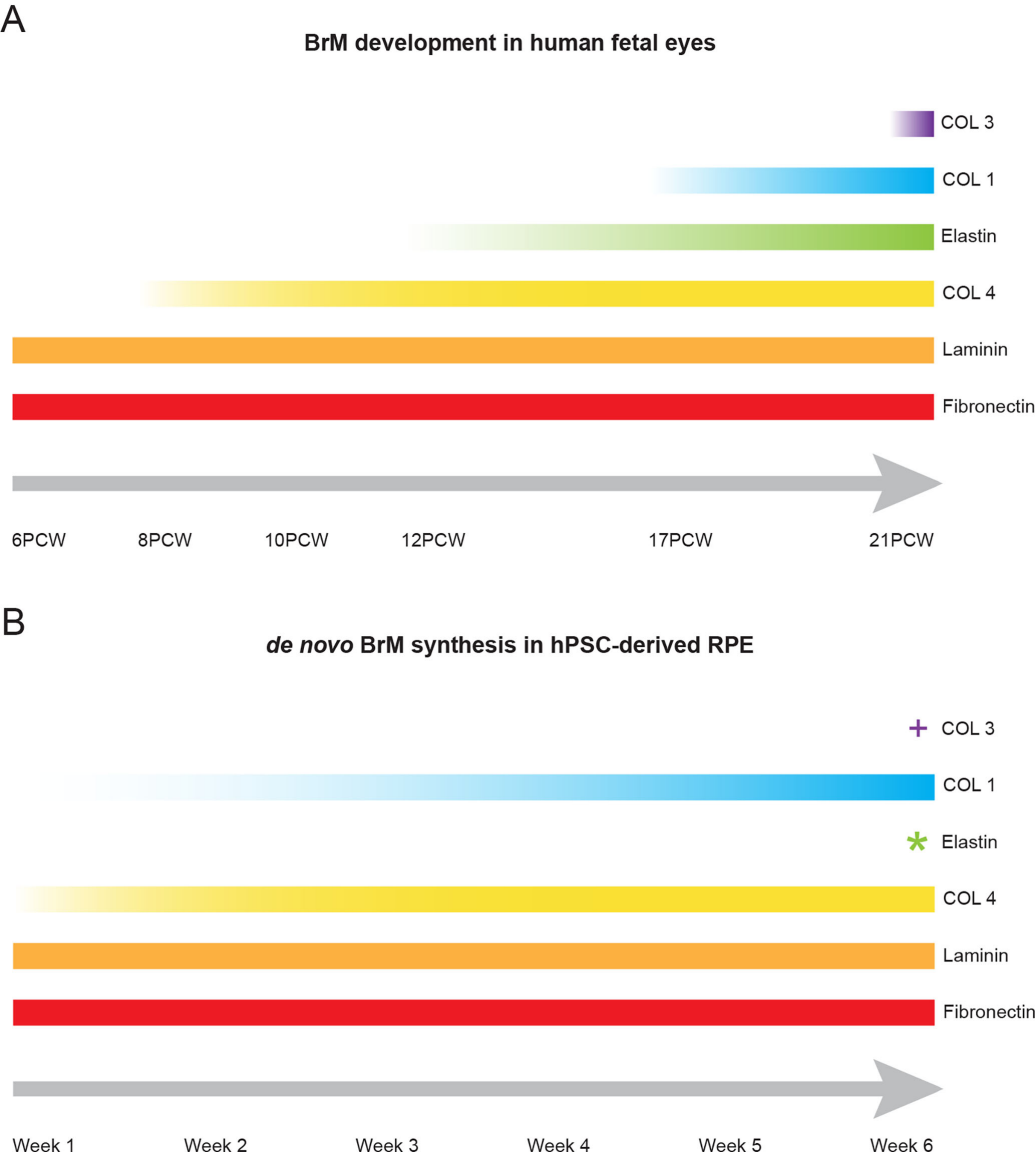
Schematic illustrating the temporal order that BrM-associated ECM proteins were detected in (**A**) human fetal retina and (**B**) hPSC-derived RPE. **^+^** Type III collagen is present in hPSC-derived RPE at all timepoints but was not localized to a de novo BrM. * Elastin was detected in one hPSC-derived RPE sample at 6 weeks, indicating elastin deposition may begin around this time.

The presence of BrM-associated proteins in hPSC-derived RPE cultures was assessed at weekly timepoints for up to 6 weeks post-seeding (Figs. 6, 7). Laminin was located basal to the RPE monolayer in the form of a defined basement membrane as early as 1 week after plating and in all subsequent timepoints (see Fig. 6A). Despite *ELN* upregulation at the RNA level, elastin protein was undetectable in hPSC-derived RPE cultures at all timepoints (see Fig. 6B), except for 1 sample at the week 6 timepoint, where elastin was evident in an isolated area (see Fig. 6Bvii). One week after plating, scattered type IV collagen deposits were observed, which, from week 2 onward, formed into a fibrous membrane-like structure located basal to the RPE monolayer (see Fig. 6C). Like type IV collagen, type I collagen was visible from week 1 as punctate deposits throughout the cell monolayer but did not form a continuous layer until the fourth week (see Fig. 7A). Type III collagen was detected in RPE cultures in all timepoints assessed but was not located basal to the RPE (see Fig. 7B), as in adult RPE. A basement membrane containing fibronectin was detected from 1-week post-seeding, and in all subsequent timepoints (see Fig. 7C). No fluorescent signal was detected in the negative controls (Supplementary Fig. S5). These results demonstrate the temporal order in which these BrM-associated proteins are synthesized in hPSC-derived RPE cultures is largely consistent with the order observed in native human fetal retina (Fig. 8).

## Discussion

Protocols for the derivation of hPSC-derived RPE are well-established and have many potential applications in furthering our understanding of human ocular development, disease modeling, and in cell replacement therapies. In vivo, BrM is formed by, and is essential for maintaining, healthy RPE; without it the death of RPE and, subsequently, photoreceptors occur resulting in visual loss.^23^^,^^51^ Despite its importance, and significant investigations into what happens to it in pathological conditions,^2^^,^^12^^,^^14^^,^^52^^,^^53^ surprisingly little is known about the formation of BrM in human development. Moreover, we do not know how accurately this process is replicated by hPSC-derived RPE.

The hPSC-derived in vitro models of ocular function are likely to become of increasing importance since the FDA Modernization Act 2.0 (12/2022) removed the requirement for animal testing of novel medicines prior to clinical studies in humans, and there is increasing interest in developing more complex models of organoids involving the incorporation of other structures including the vasculature, RPE, and the immune system.^22^ Ensuring that these structures replicate normal human development as accurately as possible becomes of paramount importance.

Similarly, RPE transplantation, either as a cell suspension or as a monolayer, is gaining significant traction as part of a comprehensive cell replacement strategy for the treatment of complex retinal degenerative diseases, like AMD. Whereas some existing strategies for RPE sheet transplantation involve non-degradable scaffolds to provide BrM-like support, prolonged presence of these foreign objects can have potentially adverse effects. Therefore, there is interest in understanding whether donor cells can form their own BrM-like structure, and, if not, what precise additional support they may require for survival and integration into the host chorioretinal complex. Here, to the best of our knowledge, we present the most comprehensive temporal characterization of human BrM formation to date and compared this to the synthesis of BrM proteins by hPSC-derived RPE.

The earliest ECM proteins to be detected in human fetal tissue were laminin and fibronectin, consistent with previous findings.^54^ Laminin is located in the two outermost layers of BrM: the basal lamina of the RPE and the basal lamina of the choriocapillaris.^2^^,^^4^ A clearly defined layer directly adjacent to the RPE was visible in all timepoints, whereas the second interrupted layer began to appear from 10 PCW, the interruptions identifying the fenestrations within the choriocapillaris. This indicates that the formation of the basal lamina of the RPE precedes that of the choriocapillaris, consistent with non-human mammalian BrM development,^16^^,^^55^ whereas type IV collagen, which is present in the basal laminae of both the RPE and choriocapillaris,^2^^,^^3^ was only evident from 8 PCW. In contrast to reports examining murine BrM, where the collagenous layers precede formation of the elastic layer,^55^ in human BrM, the elastic layer was demonstrably more established earlier than either of the collagenous layers, with types I and III collagen not seen until at least PCW 17. Given that retinal maturation exhibits a center-to-periphery gradient of retinal maturation we explored whether this was reflected in differences in BrM type IV collagen localization across the window of expression onset, however, no obvious differences were seen between the center and periphery in the qualitative immunofluorescence-based examination presented here, suggesting expression onset occurs across the eye in between these timepoints. To further assess the presence of a developmental gradient across the retina, a higher resolution technique, such as electron microscopy, in addition to closer intervals between developmental timepoints would be beneficial.

It must be noted that due to the scarcity of human fetal tissue across this period of development, the human fetal data presented in this study are from a single sample per timepoint and our conclusions are, accordingly, qualified. Although we cannot be certain that no ocular genetic mutations are present in the tissue, no chromosomal abnormalities were detected, and the samples were assessed as anatomically normal. Moreover, the trend and order of expression across the fetal samples and hRPE were broadly consistent (except elastin), lending weight to these being a fair reflection of BrM development. Further support comes from comparison with scRNAseq datasets comparing differential gene expression in human fetal RPE between PCW 12 and PCW 20, in which *COL1A1*, *COL1A2*, and *COL3A1* were in the top 20 most significantly upregulated genes (of >1000 examined; Collins et al., 2023), consistent with the onset of detectable types I, III, and IV collagen protein expression shown here. Taken together with the published literature, our findings indicate that the broad order in which the layers of the human BrM arise are: (1) basal lamina of the RPE, (2) basal lamina of the choriocapillaris, (3) elastic layer, and (4) inner and outer collagenous layers.

We next sought to assess the ability of hPSC-derived RPE to synthesize a de novo BrM in culture, examining gene and protein expression. Except for elastin, the temporal order in which the proteins assessed in this study were detected in hPSC-derived cultures largely followed that observed in human fetal development. However, the distinct layers we see in vivo were not detected in hPSC-derived RPE. This may be due to the absence of the central elastic layer, resulting in the outer layers being less well distinguished. Notably, RNA expression for *LAMA5*, *COL1A1*, *COL3A1*, *FN1*, and *ELA* did not change significantly between 2 and 6 weeks in vitro, even though immunolabeling over the same period increased, consistent with the idea that basement membranes are continuously turned over,^56^ albeit that some proteins are initially laid down more slowly than others. It should be noted that hPSC-derived RPE cultures assessed here were grown on coated glass, and that the basement membrane deposition may differ on substrates more similar to native BrM, such as porous well-inserts.

Interestingly, whereas the *ELN* gene is significantly upregulated in hPSC-derived RPE from week 2 onward, we failed to detect elastin protein with the exception of one area in a single culture. This indicates that hPSC-derived RPE has the potential to synthesize elastin protein, but additional factors may be required for more consistent synthesis or stable deposition of the protein. Likewise, type III collagen was detected in hPSC-derived RPE cultures, but it was not localized to the forming basement membrane of these cells. One candidate factor that may play a role in the synthesis of elastin and other BrM-associated proteins is lysyl oxidase (LOX), an angiocrine factor released by choroidal endothelial cells.^28^ Inhibition of LOX activity in mice has been found to result in poor BrM assembly,^28^ and BrM elastin formation is significantly impaired in LOX-like protein 1-deficient mice.^57^ In addition, LOX is known to be upregulated in diabetic retinopathy,^58^ which is a disease also associated with increased ECM crosslinking and resultant BrM thickening.^59^ This, along with improved BrM formation in endothelial co-culture,^27^^,^^28^^,^^31^^,^^32^ suggests that the presence of the choroid may be required for BrM elastin deposition. With respect to transplantation, where an RPE monolayer is grafted into the sub-retinal space, upon correct placement the basal side of the donor RPE would be adjacent to the host choroid. Therefore, angiocrine factors released by the host choroidal endothelial cells may be sufficient to catalyze de novo ECM protein synthesis by donor RPE post-transplantation, including those of elastin and type III collagen. Alternatively, it may be possible to add relevant factors to the graft pre-transplantation to ensure optimal graft condition at the time of transplantation.

The findings from this study reveal the likely temporal profile of both human fetal BrM development and de novo hPSC-derived RPE membrane synthesis in vitro. We show that protein deposition begins in the first week post-seeding of hPSC-derived RPE, and the order of layer formation broadly reflects human BrM development, although elastin protein is absent from the de novo synthesized BrM-like membrane. It is important to keep in mind that the methods of RPE differentiation used here (and in many other protocols) involve isolating RPE that has already started to differentiate and then isolating and passaging further (two times, in the case of this study) in order to expand cell numbers prior to examining the order in which BrM membrane proteins are laid down, rather than observing it from the first commitment to an RPE fate. It is likely that some of these basement membrane genes are already starting to be expressed in the isolated cells during the expansion period. Nonetheless, the order of deposition, upon differentiation, still appears to be broadly comparable with de novo expression in fetal retina. In addition to continuing to support the use of hPSC-derived RPE as a model system for exploring RPE function and disease, these findings will also help inform the design of tailored scaffolds to facilitate RPE cell-replacement to treat retinal degeneration. For instance, considerations of the condition of the host BrM (likely thickened and stiffer as a result of age and disease) might be taken into account; debridement to remove the diseased tissue may be necessary, and, upon transplant, could be replaced with an artificial scaffold, which, as it degrades, may be replaced by a donor RPE-synthesized membrane.

## Supplementary Material

Supplement 1
