# Supplementary material for: Characterization of Bruch's Membrane Formation in Human Fetal Retina and De Novo Membrane Synthesis by hPSC-Derived Retinal Pigment Epithelium
Source: Invest Ophthalmol Vis Sci. 2025 Jun 11;66(6):40. doi: 10.1167/iovs.66.6.40 (PMC12166504; doi:10.1167/iovs.66.6.40)
Supplement: Supplement 1 [file iovs-66-6-40_s001.pdf]

Supplementary Methods

Enzyme-linked immunosorbent assay (ELISA)

hPSC-derived RPE were differentiated and cultured as described above. At the second passage, cells were seeded onto vitronectin coated ThinCerts® (Greiner Bio-One, UK). At 6 weeks post-seeding, and after 72 hours without media change, media was collected from the separate apical and basolateral compartments. Concentration of vascular endothelial growth factor (VEGF) in the media samples was assessed using a Human VEGF DuoSet ELISA kit (Bio-techne, MN) as per the manufacturer's instructions. Optical density was measured using a microplate reader. Relative concentrations of VEGF in the media were calculated from optical densities using a standard curve.

Antibody	Dilution	Company	Catalogue Number
Elastin	1:100	ThermoFisher	MA1-27129
Laminin	1:100	Abcam	ab11575
Fibronectin	1:100	ThermoFisher	CSI 005-17-02
Collagen I	1:100	ThermoFisher	MA1-26771
Collagen III	1:100	ThermoFisher	PA5-34787
Collagen IV	1:100	Bio-Rad	2150-1470
RPE65	1:100	ThermoFisher	MA1-16578
ZO-1	1:200	ZO-1	402200
Bestrophin	1:200	Merck	MAB5466
Ezrin	1:200	Cell Signalling Technology	3145S

Supplementary Table 1. Antibodies

Gene	Forward	Reverse
ELN	CTGGTCTCGGAGCCCTTG	CCTCCGGGAACTGGCTTA
LAMA5	ACCTGTCCAGCCCAGGAT	CTGCCCTCCTCTCGCAC
FN1	CCGCCGAATGTAGGACAAG	GCCCATGAGATGGTTGTCTG
COL1A1	AAATGGAGCTCCTGGTCAGA	GTAGCACCATCATTTCCACGA
COL3A1	GAGGATGGTTGCACGAAACA	TGATCAGGACCACCAATGTCA
COL4A5	TCTCCTGAGAGACCGGCTT	AGAGAGCTAGAGCCGGAGAG
RPE65	ATCCTGCTGGTGGTTACAAGA	AAAGAGTCCTGGCCCACATC
MITF	CACCATCAGCAACTCCTGTC	GCTCTTGCTTCAGACTCTGTG
BEST1	ACAAGCCAAGTGGCTAATGC	CGTGAGGGCCAGCCTATAAA
BACT	GCACCCAGCACAATGAAGAT	TCGTCATCCTGCTTGCT
GAPDH	TTGCCCTCAACGACCACTTT	TGGTCCAGGGGTCTTACTCC

Supplementary Table 2. Primers

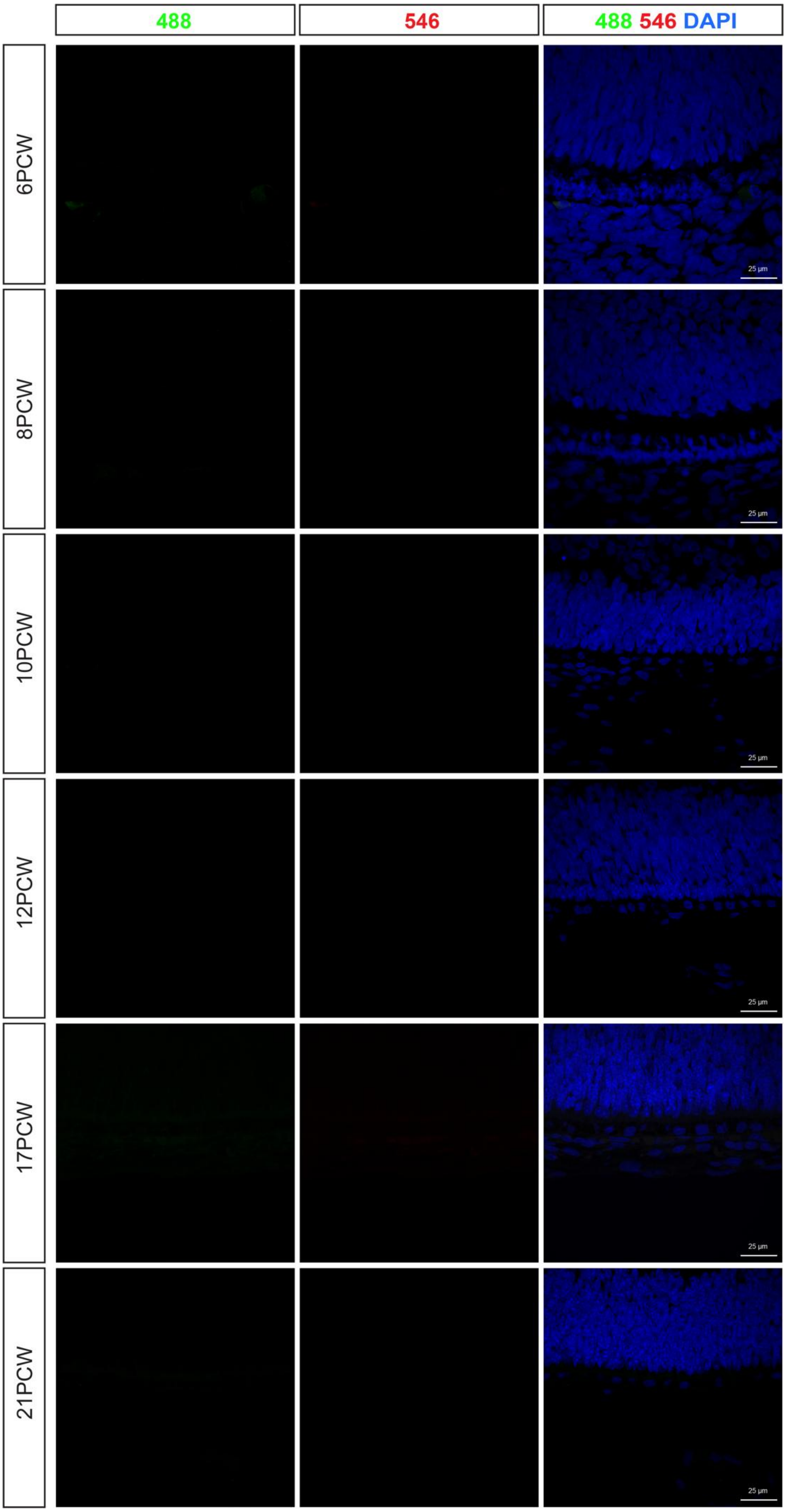
488
546
488 546 DAPI
6PCW
8PCW
10PCW
12PCW
17PCW
21PCW
25 µm

Supplementary Figure 1. No-primary negative control for immunohistochemistry of human foetal sections Immunohistochemistry without primary antibody conducted on cryosections through human fetal retina. Secondary antibodies donkey anti-mouse 488 (*green*) and donkey anti-rabbit 546 (*red*; Alexa Fluor™, 1:200, ThermoFisher™, UK) were used and nuclei were counter stained with DAPI (*blue*). Images show confocal maximum intensity projections (MIPs) of a z-stack ~1µm thick, taken from the superior retina in the equatorial region. Scale bars, 25µm.

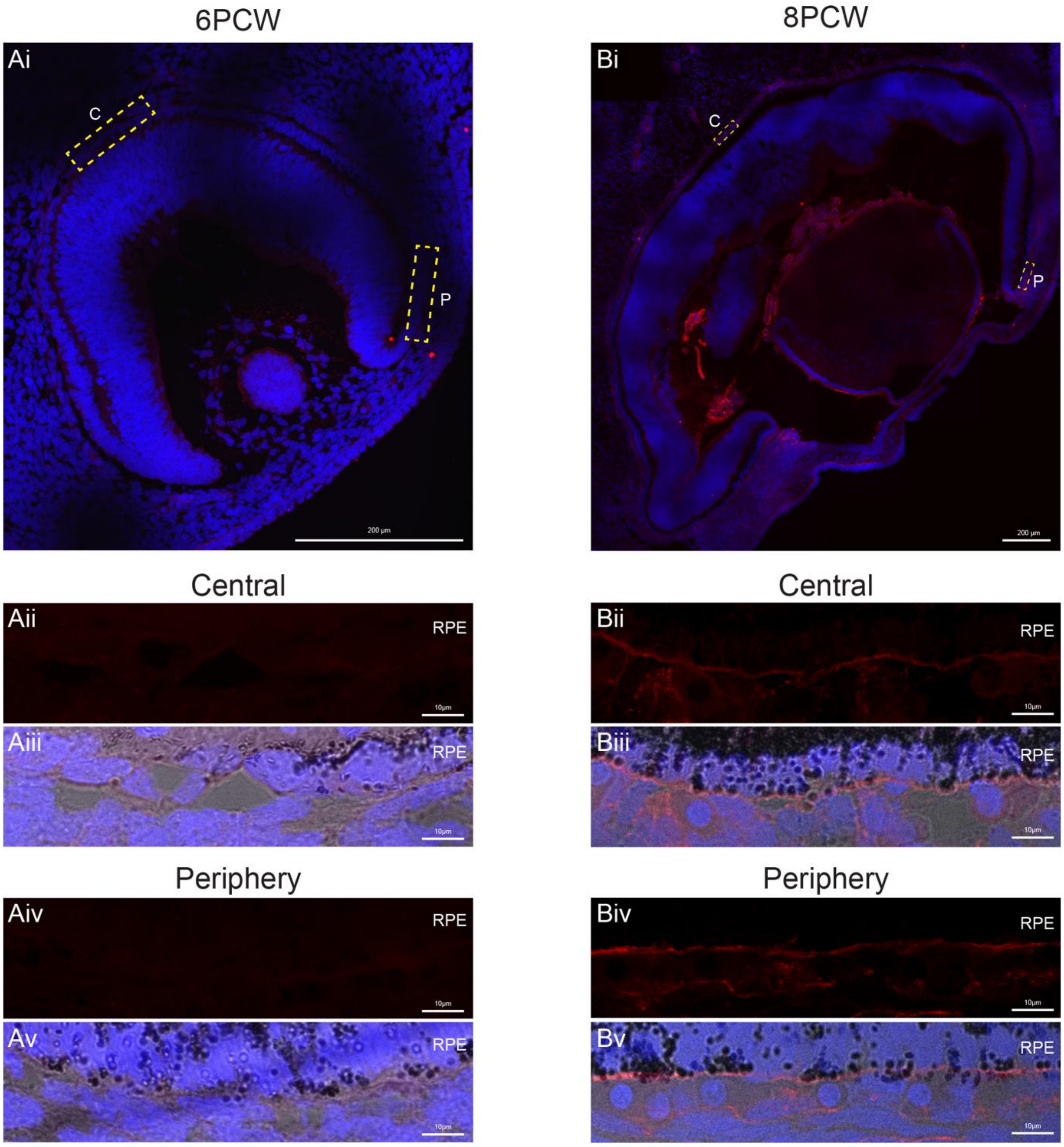

Supplementary Figure. 2. Comparison of central vs peripheral COL4 staining in PCW6 and PCW8 foetal retina Cryosections through human fetal retina and immunolabelled for collagen IV. (A) Immunostaining for Type IV collagen *(red)* at 6PCW shows labelling an absence of Type IV collagen from both the centre and the periphery. (B) Immunostaining for Type IV collagen *(red)* at 8PCW reveals labelling basal to the RPE in both the central and peripheral retina. Images show confocal MIPs of a z-stack ~1μm thick, taken from the central or peripheral retina. C – central retina; P – peripheral retina; BrM – Bruch's membrane; RPE– retinal pigment epithelium; BF - Brightfield; PCW – post-conception weeks; Nuclei were counter stained with DAPI (*blue*).

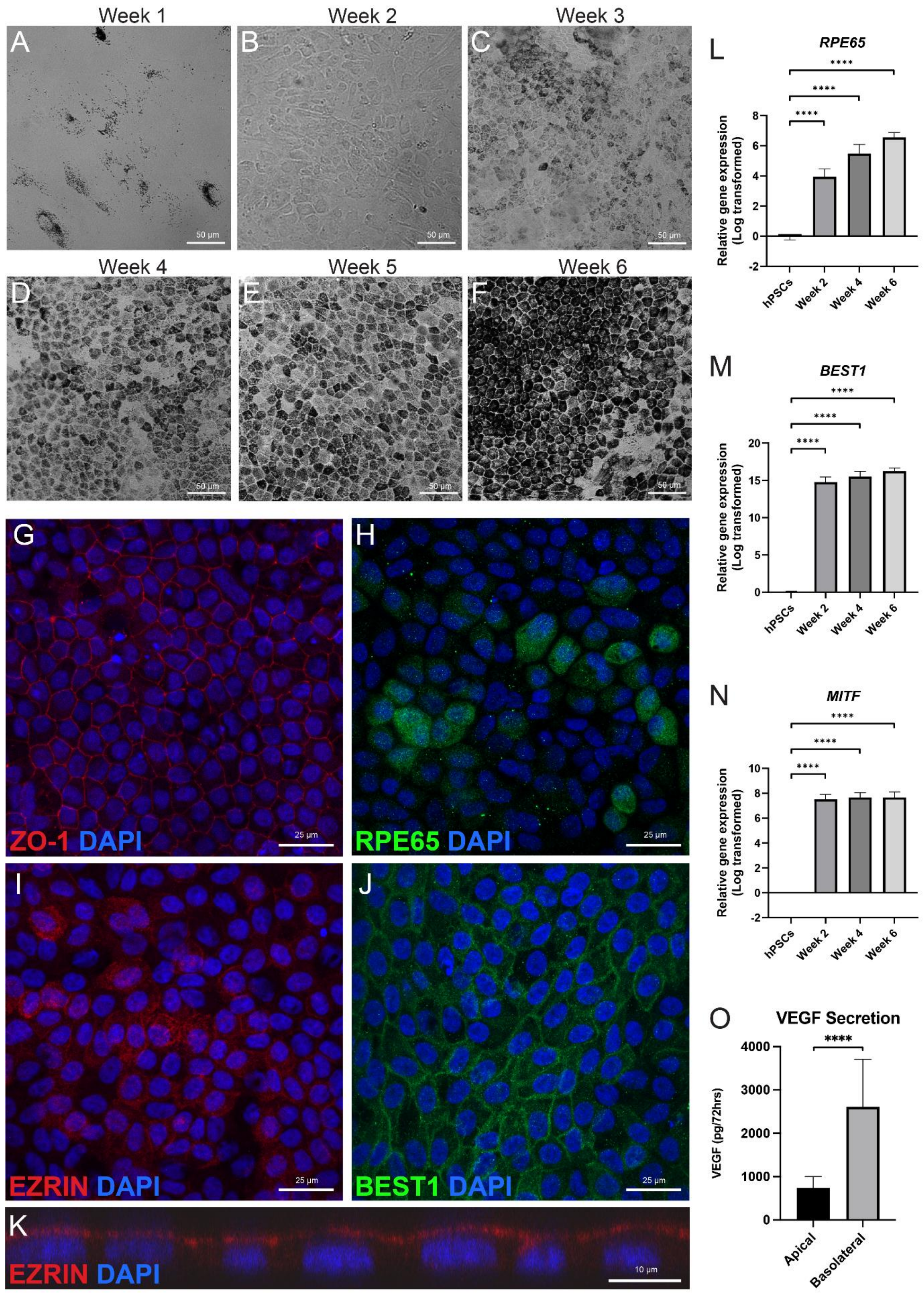

Week 1
Week 2
Week 3
Week 4
Week 5
Week 6
A
B
C
D
E
F
50 µm
G
H
I
J
K
ZO-1 DAPI
RPE65 DAPI
EZRIN DAPI
BEST1 DAPI
25 µm
10 µm
L
RPE65
M
BEST1
N
MITF
Relative gene expression (Log transformed)
hPSCs
Week 2
Week 4
Week 6
****
O
VEGF Secretion
VEGF (pg/72hrs)
Apical
Basolateral

Supplementary Figure. 3. Characterisation of hPSC-derived RPE (A)-(F) Representative brightfield images of hPSC-derived RPE over a 6-week period. Images show an increase in pigmentation, confluence and cobblestone-like morphology throughout the 6-week period. (G)-(J) Representative confocal maximum projection images (MIPs) of a z-stack ~15µm thick showing immunostaining for (G) ZO-1, (H) RPE65, (I) Ezrin, and (J) Bestrophin at week 6. (K) Simulated cross-sections using XZ orthogonal projections show immunostaining for Ezrin at the apical surface. (L)-(N) Relative expression of *RPE65*, *BEST1*, and *MITF* in hPSC-RPE at weeks 2, 4 and 6 post-seeding, relative to undifferentiated hPSCs. One-way ANOVA with Dunnett's multiple-comparison test. (O) Concentration of vascular endothelial growth factor (VEGF) detected by ELISA in apical and basolateral culture media in week-6 hPSC-derived RPE cultures. Unpaired t-test, *$p<0.05$, **$p<0.01$, ***$p<0.001$, ****$p<0.0001$.

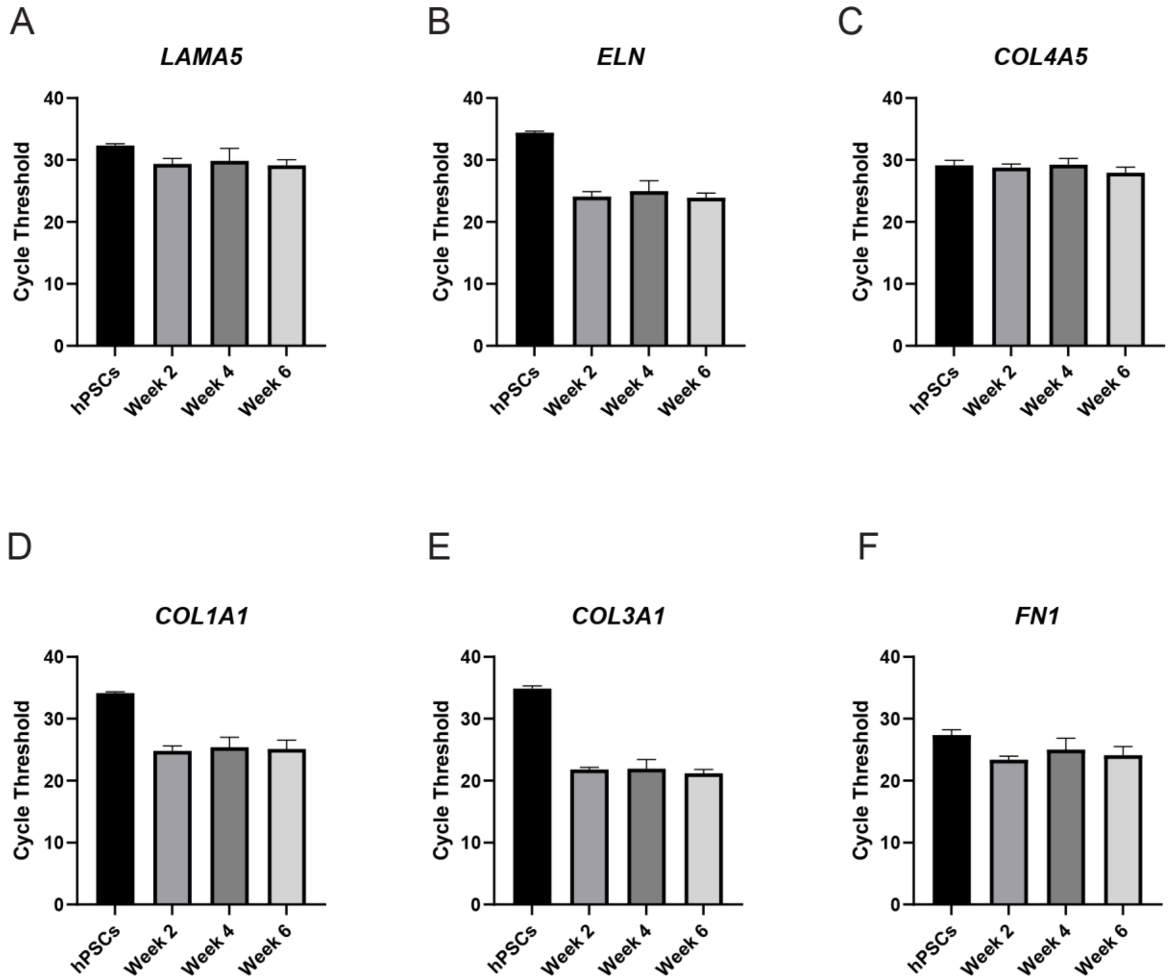

Supplementary Figure 4. Cycle threshold qPCR data (A)-(F) Cycle threshold values from qPCR analysis of (A) *LAMA5*, (B) *ELN*, (C) *COL4A5*, (D) *COL1A1*, (E) *COL3A1*, and (F) *FN1 expression* in undifferentiated hPSCs and hPSC-derived RPE at weeks 2, 4, and 6 post-seeding.

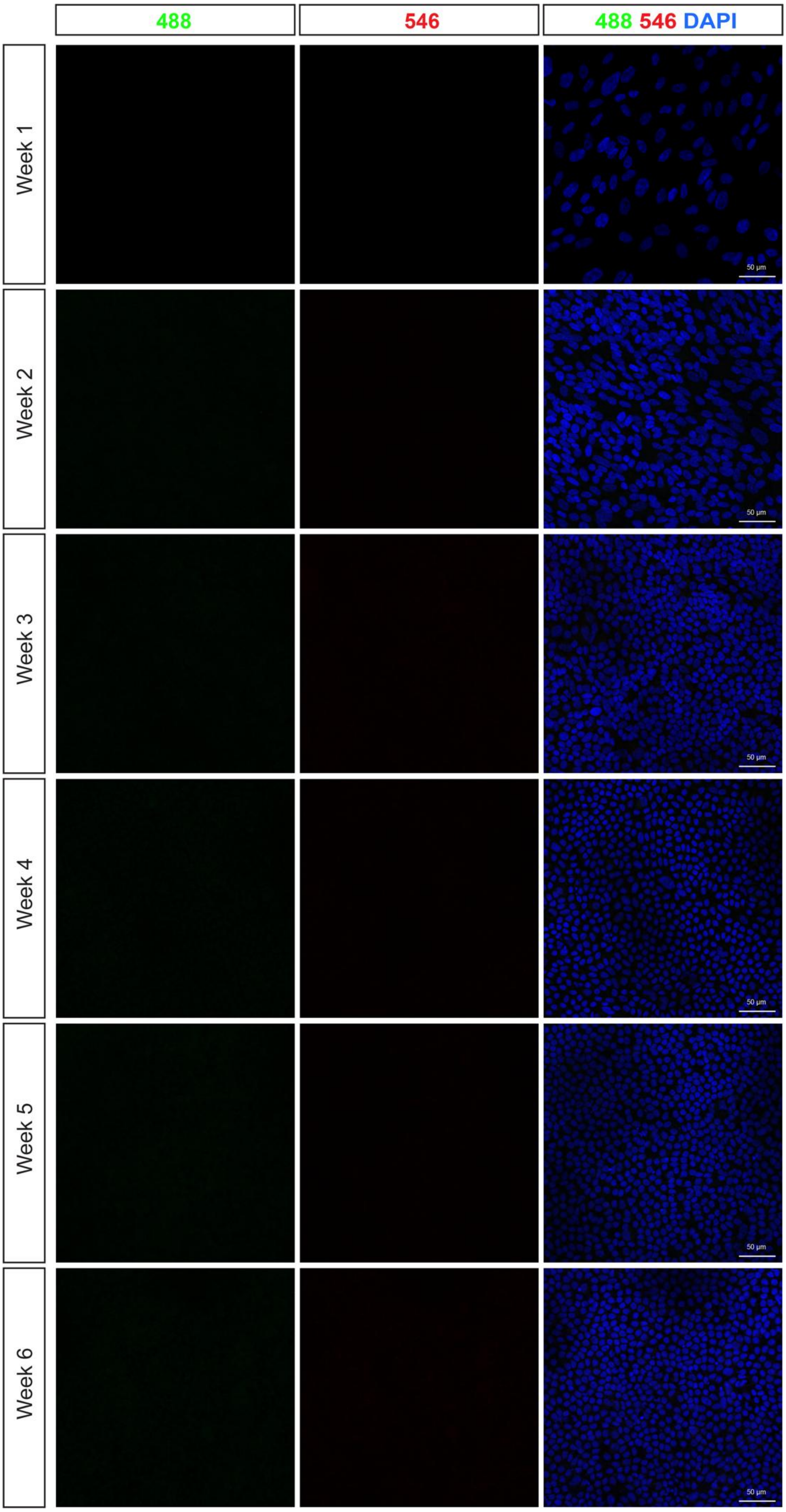
488
546
488 546 DAPI
Week 1
Week 2
Week 3
Week 4
Week 5
Week 6
50 µm

Supplementary Figure 5. No-primary negative control for immunocytochemistry of hPSC-derived RPE cultures Immunocytochemistry without primary antibody conducted on hPSC-RPE over a 6-week culture period. Secondary antibodies donkey anti-mouse 488 (*green*) and donkey anti-rabbit 546 (*red*; Alexa Fluor™, 1:200, ThermoFisher™, UK) were used and nuclei were counter stained with DAPI (*blue*). Images show confocal maximum intensity projections (MIPs) of a z-stack ~1µm thick, taken from the superior retina in the equatorial region. Scale bars, 50µm.
